# An enhanced IL17 and muted type I interferon nasal epithelial cell state characterizes severe COVID-19 with fungal coinfection

**DOI:** 10.1128/spectrum.03516-23

**Published:** 2024-04-30

**Authors:** Carly G. K. Ziegler, Anna H. Owings, Michelle Galeas-Pena, Samuel W. Kazer, Vincent N. Miao, Andrew W. Navia, Ying Tang, Joshua D. Bromley, Peter Lotfy, Meredith Sloan, Hannah Laird, Haley B. Williams, Micayla George, Riley S. Drake, Yilianys Pride, George E. Abraham, Michal Senitko, Tanya O. Robinson, Gill Diamond, Michail S. Lionakis, Alex K. Shalek, Jose Ordovas-Montanes, Bruce H. Horwitz, Sarah C. Glover

**Affiliations:** 1 Program in Health Sciences & Technology, Harvard Medical School & MIT, Boston, Massachusetts, USA; 2 Ragon Institute of MGH, MIT, and Harvard, Cambridge, Massachusetts, USA; 3 Broad Institute of MIT and Harvard, Cambridge, Massachusetts, USA; 4 Harvard Graduate Program in Biophysics, Harvard University, Cambridge, Massachusetts, USA; 5 Institute for Medical Engineering & Science, Massachusetts Institute of Technology, Cambridge, Massachusetts, USA; 6 Department of Medicine, University of Mississippi Medical Center, Jackson, Mississippi, USA; 7 Department of Medicine, Section of Gastroenterology and Hepatology, Tulane University School of Medicine, New Orleans, Los Angeles, USA; 8 Program in Immunology, Harvard Medical School, Boston, Massachusetts, USA; 9 Division of Emergency Medicine, Boston Children’s Hospital, Boston, Massachusetts, USA; 10 Department of Chemistry, Massachusetts Institute of Technology, Cambridge, Massachusetts, USA; 11 Division of Gastroenterology, Hepatology, and Nutrition, Boston Children’s Hospital, Boston, Massachusetts, USA; 12 Department of Microbiology, Massachusetts Institute of Technology, Cambridge, Massachusetts, USA; 13 Division of Digestive Diseases, University of Mississippi Medical Center, Jackson, Mississippi, USA; 14 Division of Pulmonary, Critical Care, and Sleep Medicine, University of Mississippi Medical Center, Jackson, Mississippi, USA; 15 Department of Oral Immunology and Infectious Diseases, University of Louisville, Louisville, Kentucky, USA; 16 Fungal Pathogenesis Section, Laboratory of Clinical Immunology and Microbiology (LCIM), National Institute of Allergy and Infectious Diseases (NIAID), Bethesda, Maryland, USA; 17 Koch Institute for Integrative Cancer Research, Massachusetts Institute of Technology, Cambridge, Massachusetts, USA; 18 Harvard Stem Cell Institute, Cambridge, Massachusetts, USA; 19 Center for Immunology and Microbial Research, Department of Cell & Molecular Biology, University of Mississippi Medical Center, Jackson, Mississippi, USA; Geisel School of Medicine at Dartmouth, Lebanon, New Hampshire, USA

**Keywords:** SARS-CoV-2, COVID-19, human, nasal mucosa, epithelial immunity, *Candida*, fungal infection, IL17, cytokine, interferon, anti-viral, scRNA-seq

## Abstract

Recent case reports and epidemiological data suggest that fungal infections represent an underappreciated complication among people with severe COVID-19. However, the frequency of fungal colonization in patients with COVID-19 and associations with specific immune responses in the airways remain incompletely defined. We previously generated a single-cell RNA-sequencing data set characterizing the upper respiratory microenvironment during COVID-19 and mapped the relationship between disease severity and the local behavior of nasal epithelial cells and infiltrating immune cells. Our previous study, in agreement with findings from related human cohorts, demonstrated that a profound deficiency in host immunity, particularly in type I and type III interferon signaling in the upper respiratory tract, is associated with rapid progression to severe disease and worse clinical outcomes. We have now performed further analysis of this cohort and identified a subset of participants with severe COVID-19 and concurrent detection of *Candida* species-derived transcripts within samples collected from the nasopharynx and trachea. Here, we present the clinical characteristics of these individuals. Using matched single-cell transcriptomic profiles of these individuals’ respiratory mucosa, we identify epithelial immune signatures suggestive of IL17 stimulation and anti-fungal immunity. Further, we observe a significant expression of anti-fungal inflammatory cascades in the nasal and tracheal epithelium of all participants who went on to develop severe COVID-19, even among participants without detectable genetic material from fungal pathogens. Together, our data suggest that IL17 stimulation—in part driven by *Candida* colonization—and blunted interferon signaling represent a common feature of severe COVID-19 infection.

**IMPORTANCE:**

In this paper, we present an analysis suggesting that symptomatic and asymptomatic fungal coinfections can impact patient disease progression during COVID-19 hospitalization. By looking into the presence of other pathogens and their effect on the host immune response during COVID-19 hospitalizations, we aim to offer insight into an underestimated scenario, furthering our current knowledge of determinants of severity that could be considered for future diagnostic and intervention strategies.

## INTRODUCTION

Infection with SARS-CoV-2, the virus that causes COVID-19, can lead to severe viral pneumonitis and the development of acute respiratory distress syndrome (1, 2). Severe COVID-19 is characterized by peripheral immune dysregulation, and we and others have previously demonstrated blunted interferon responses within the nasal mucosa of patients with severe COVID-19 (3–5). Recent case reports and retrospective cohort studies suggest that secondary infection with fungal pathogens may be a significant contributor to morbidity and mortality in patients with severe COVID-19 (6–11).

The frequency of fungal colonization of the airways in patients with severe COVID-19 and its potential impact on local mucosal immunity remain unknown (9, 12–15). IL17, released by CD4 T cells and innate lymphocytes, is a key effector cytokine that coordinates mucosal anti-fungal immunity among other adaptive and innate leukocytes, granulocytes, and mucosal stroma (16–19). Recent work has uncovered complex interactions between IL17-driven inflammation, type 1 interferon responses, and susceptibility to fungal pathogens; however, the effect of fungal colonization and anti-fungal immune responses during cooccurrent SARS-CoV-2 infection has yet to be explored (20). Here, using a previously published data set derived from a cohort of individuals acutely infected with SARS-CoV-2, we directly assessed cooccurrent fungal colonization in the airways of patients with severe COVID-19 and examined pathways associated with anti-fungal immunity (3).

## RESULTS

**Fig 1:**
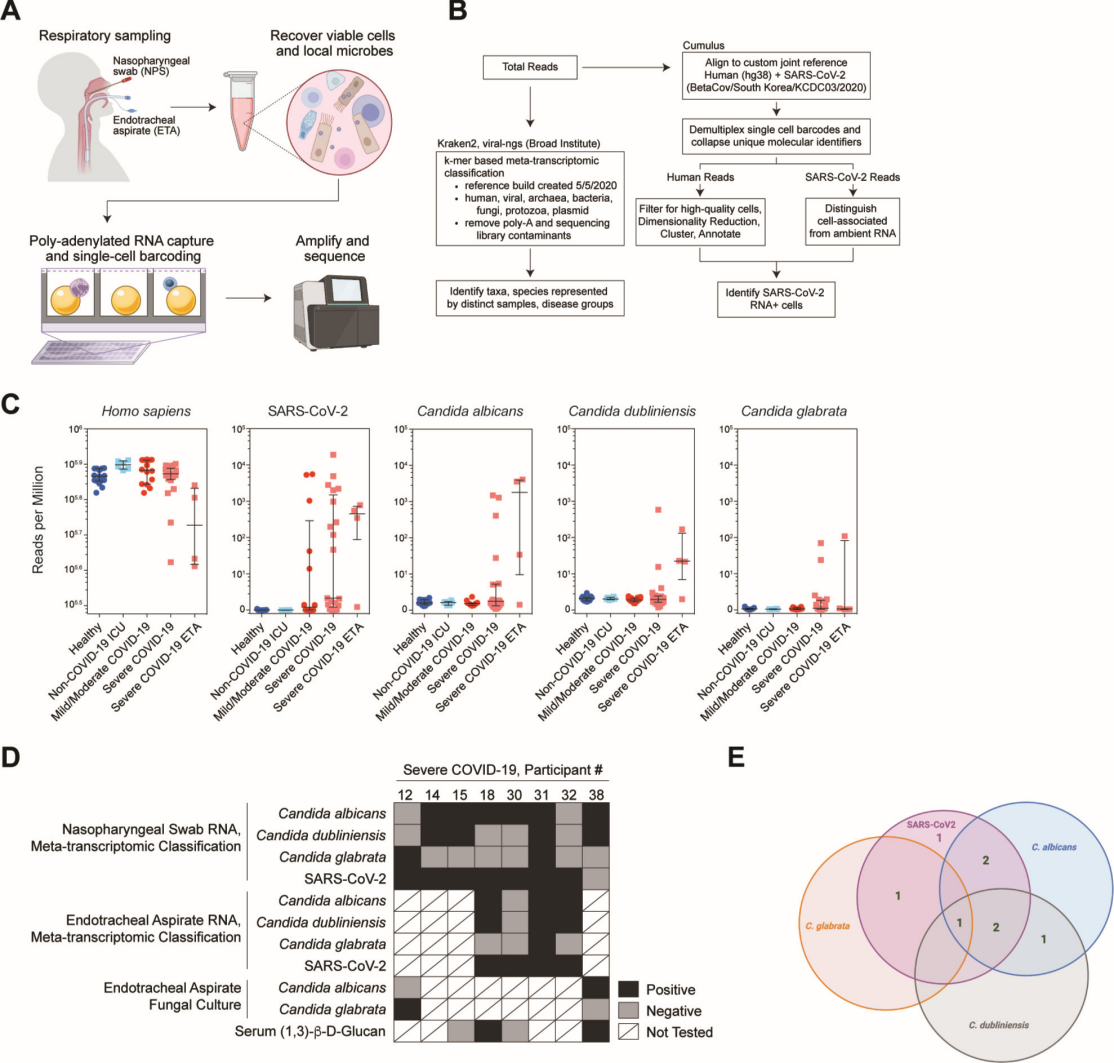
Codetection of host single-cell transcriptome with intracellular and microenvironmental pathogen-derived genomic material. (**A**) Schematic of biological sample processing pipeline. (**B**) Schematic of computational pipeline for each sample. (**C**) Abundance of human, SARS-CoV-2, and *Candida* spp. by participant and disease group as defined by meta-transcriptomic classification. *N* = 56 participants. Lines represent median ±interquartile range. (**D**) Summary of results from sequencing, fungal culture, and serum (1,3)-β-D-glucan assays from eight participants with detectable *Candida* species reads by nasopharyngeal swab or endotracheal aspirate. (**E**) Graphical summary of COVID-19 and *Candida* sp. infection among severe COVID-19 cohort.

We had previously described a cohort of 58 individuals—56 of which are further characterized here—including 15 healthy participants, 35 individuals diagnosed with acute COVID-19, and 6 intubated patients that were negative for SARS-CoV-2 (3) The two excluded individuals from the original cohort had recovered from COVID-19 (“convalescent COVID-19”) and, therefore, did not represent acute infection. We were assessing phenotypes of respiratory epithelia early following infection. Nasopharyngeal (NP) swabs obtained from these patients were employed in a cross-sectional study of the nasal respiratory cellular composition using single-cell RNA sequencing (scRNA-seq) (Fig. 1A and B). Patients in this cohort with COVID-19 were sampled within 9 days of hospital admission (median: hospital day 2), which we estimated was within 2 weeks of initial respiratory symptoms. Full cohort demographic data and findings relating to the cellular composition, behaviors, and response to SARS-CoV-2 infection between disease groups can be found in our prior manuscript (3). Eight patients from this cohort had detectable *Candida* species reads from nasopharyngeal swabs or endotracheal aspirate (ETA) samples by meta-transcriptomic analysis. *Candida* species infection was confirmed in three of these patients by fungal culture and/or serum (1,3)-β-D-glucan. We found no difference in the frequency of antibiotic treatment nor corticosteroid exposure among patients from mild/moderate COVID-19, non-COVID-19 intensive care unit (ICU) intubated controls, severe COVID-19 *Candida* negative, or severe COVID-19 *Candida* positive.

We had previously applied a meta-transcriptomic taxonomic classification analysis to each sample to assign both cell-associated and ambient/extracellular sequencing reads to a reference database of human and microbial genomes (generated on 5 May 2020 from the NCBI Reference Sequence Database including archaeal, bacterial, viral, protozoan, and fungal genomes) (21, 22). This approach, in addition to direct reference-based alignment, enabled us to quantify respiratory abundances of SARS-CoV-2 and connect viral abundances to the cellular sources of viral replication, as well as concurrent epithelial host responses (Fig. 1B). Here, we describe further analysis of the data generated from these nasopharyngeal samples, as well as additional data generated from matched ETAs obtained from four of the individuals in the original cohort with severe COVID-19.

### Candida species coinfection was limited to severe COVID-19 patients

Using meta-transcriptomic alignment of scRNA-seq data, we identified additional high-abundance microbial taxa across healthy participants and those with COVID-19 including common commensal microbes such as *Cutibacterium acnes, Malassezia restricta*, and *Staphylococcus aureus* (Fig. S1A; Table S1). After SARS-CoV-2, the second-most abundant microbe detected was *Candida albicans,* which was detected on six NP samples obtained from patients with severe COVID-19 (Fig. 1C through E; Fig. S1A). For a subset of patients with PCR-positive SARS-CoV-2 and clinical evidence of COVID-19 (both requirements for inclusion into our “COVID-19” group), there are no reads that align with SARS-CoV-2. However, SARS-CoV-2 RNA was detected from scRNA-seq libraries in 80% (28/35) of participants diagnosed with COVID-19. This proportion is in accordance with larger studies that assess the likelihood of a positive Real Time Polymerase Chain Reaction (RT-PCR) result by nasopharyngeal sampling. In one meta-analysis by Mallett et al., 89% of RT-PCR results were positive within 4 days of symptom onset for patients with eventual confirmed COVID-19, and 81% of RT-PCR tests remained positive within the first 4 days of hospital admission (23). Further, we have assessed for concordance between Kraken2-based meta-transcriptomic capture as well as direct alignment against a SARS-CoV-2 reference sequence (Fig. S1C).

We also identified high levels of *Candida glabrata* in NP samples from two patients with severe COVID-19 and *Candida dubliniensis* in four samples. All samples that were positive for *C. glabrata* or *C. dubliniensis* were also positive for *C. albicans* with the exception of the NP sample obtained from COVID-19 participant 12 (Fig. 1D and E). *Candida albicans* was also detected in three of four ETA samples obtained from patients with severe COVID-19. For one patient (COVID-19 participant 32), *C. albicans* was detected via ETA but was not detected on their matched NP swab. All three ETA samples that were positive for *C. albicans* were also positive for *C. dubliniensis*, and one of these was also positive for *C. glabrata* (Fig. 1C through E; Fig. S1A and B). Notably, no *Candida* spp. or other fungal pathogens were detected within samples obtained from healthy individuals, those obtained from individuals with mild/moderate COVID-19, or those obtained from SARS-CoV-2 negative intubated patients in the intensive care unit with severe respiratory failure due to alternative causes. Thus, all *Candida* spp. reads were detected among patients who developed severe COVID-19 requiring intubation and mechanical ventilation (WHO severity score of 6–8). Nearly all NP or ETA samples that were positive for *Candida* spp. were collected within 1 week of hospital admission (Fig. S2A). The majority (6/8) had been intubated for at least 1 day, and 5/8 had received at least 1 day of corticosteroid treatment prior to sample collection (Fig. S2A). Clinical evaluations during hospitalization were performed to search for possible fungal infection for three of the eight patients with high abundances of *Candida* spp. by meta-transcriptomic classification. COVID-19 participant 12, who had a high abundance of *Candida glabrata* RNA sampled by NP swab on hospital day 2, was intubated on hospital day 1 (Fig. S2A), and ETA fungal cultures sampled on hospital day 2 revealed growth of *Candida glabrata* (Fig. 1D). For two participants, detection of *Candida* spp.-derived RNA via NP/ETA sampling significantly preceded clinical diagnostic testing for fungal pathogens. COVID-19 participant 38, whose NP swab revealed both *Candida albicans* and *Candida dubliniensis* RNA on hospital day 8, tested positive for serum (1,3)-β-D-glucan on hospital day 14 and had *Candida albicans* growth from ETA culture on hospital day 16 (Fig. S2A). Both the NP and ETA samples from COVID-19 participant 18 obtained on hospital day 6 contained high abundances of reads classified as *C. albicans*, and *C. dubliniensis* was detected in the ETA sample. On hospital day 12, (1,3)-β-D-glucan was detected in this individual’s serum (Fig. S2A), prompting treatment with micafungin (an echinocandin anti-fungal) (24). Four out of 21 patients with severe COVID-19 had *Candida* reads on our meta-transcriptomic analysis, but only two of those were positive by serum (1,3)-β-D-glucan testing. Together, this demonstrates that for a subset of patients, there was significant clinical concern during their hospitalization to prompt additional testing to evaluate for fungal respiratory infection or fungemia.

Among those individuals, we find concordance between the detection of *Candida*-derived RNA from scRNA-seq libraries and clinical assays during their hospitalization. Additionally, we compared the proportion of patients in each subgroup in our data set receiving corticosteroids in advance or at the time of sample collection. Thirty-three percent (2/6) of patients in the intubated non-COVID-19 ICU group were receiving corticosteroids, and 0% (0/6) had detectable *Candida* reads by our analysis. Among patients with severe COVID-19 and no *Candida* species detected via meta-transcriptomic analyses, 61% (8/13) were receiving corticosteroids. Finally, 75% (6/8) of patients with severe COVID-19 and *Candida* species detected were receiving corticosteroids. Additionally, among COVID-19 patients not in the ICU (our mild/moderate COVID-19 group), 42.9% (6/14) were receiving corticosteroids, and 0% (0/14) had detectable *Candida* reads. We did not identify any significant differences in the proportion of corticosteroid treatment across any of these four groups by the chi-squared test. Although limited by sample size, our data do not suggest that corticosteroid treatment represented a major confounder in our detection of *Candida*.

The majority of COVID-19 patients with severe disease included in our analysis were treated with antibiotics during their hospitalization. The most common antibiotic combination used was azithromycin and ceftriaxone. Among patients with severe COVID-19 and no *Candida* detected, 77% (10/13) were treated with antibiotics for at least 1 day prior to sampling. Among patients with severe COVID-19 and *Candida* species detected, 88% (7/8) were treated with antibiotics for at least 1 day in advance of nasal swab.

We did not detect a difference in demographics or clinical characteristics—apart from the severity of COVID-19—between patients whose samples did or did not contain *Candida*-specific reads (Table 1; Fig. S2B through G). Twenty-eight-day mortality rates among individuals with severe COVID-19 were similar between *Candida* spp. positive vs negative groups: 62.5% (5/8) among participants with *Candida* spp. detected, compared to 84.6% (11/13) among participants without *Candida* spp. detected. Nearly all (7/8) participants with COVID-19 whose samples contained *Candida* spp.-aligning RNA were previously diagnosed with type 2 diabetes mellitus (T2DM), and 8/8 were diagnosed with chronic hypertension. Notably, although T2DM represents an independent risk factor for mucosal *Candida* colonization, we did not find *Candida* or other fungal species among individuals with T2DM within the healthy non-COVID-19 intubated or COVID-19 mild/moderate groups (25). Additionally, for individuals with recently measured HbA1c, we did not identify significant differences in the degree of glycemic control between individuals with different COVID-19 severity or by detection of *Candida*-specific reads (Fig. S2G) (26).

**TABLE 1:** Demographics and medical comorbidities of patients with severe COVID-19^*a*^

	COVID-19 severe (WHO score 6–8) *Candida* spp. negative	COVID-19 severe (WHO score 6–8) *Candida* spp. positive
Case number	23.2% (13/56)	14.3% (8/56)
Age (years)		
Minimum	28	38
Median (IQR)	63 (49)	57 (54.3)
Maximum	79	84
Sex		
Female	38.5% (5/13)	62.5% (5/8)
Male	61.5% (8/13)	37.5% (3/8)
Ethnicity		
Hispanic	7.7% (1/13)	0% (0/13)
Not Hispanic	92.3% (12/13)	100% (13/13)
Race		
Black/African American	53.8% (7/13)	75% (6/8)
White	23.1% (3/13)	25% (2/8)
American Indian	23.1% (3/13)	0% (0/8)
BMI		
Median (IQR)	29.9 (27.8)	37.0 (33.6)
Pre-existing conditions		
Diabetes	61.5% (8/13)	87.5% (7/8)
Chronic kidney disease	15.4% (2/13)	25% (2/8)
Congestive heart failure	7.7% (1/13)	0% (0/8)
Lung disorder	30.1% (4/13)	50% (4/8)
Hypertension^*b*^	69.2% (9/13)	100% (8/8)
IBD	0% (0/13)	0% (0/8)
Treatment		
Corticosteroids	61.5% (8/13)	75% (6/8)
Remdesivir	7.7% (1/13)	0% (0/8)
28-day mortality^*c*^	84.6% (11/13)	62.5% (5/8)

^*a*^ IQR, interquartile range; BMI, body mass index; IBD, inflammatory bowel disease.

^*b*^ *p = 0.023.

^*c*^ ***p < 0.0001.

In summary, we identified 0% (0/14) of patients with mild/moderate COVID-19 with *Candida* reads despite equivalent library quality characteristics. This contrasts with the 38% (8/21) of patients with severe COVID-19 with associated *Candida*-mapping reads, corresponding to *P* = 0.0118 by the Fisher exact test. Additionally, we found *Candida* reads in 0% (0/6) of patients who were intubated in the intensive care unit. Critically, this analysis is based on a limited sample size and merits further investigation with adequately powered cohorts.

### The nasal epithelium of patients with severe COVID-19 presented increased expression of IL17A-induced genes

**Fig 2:**
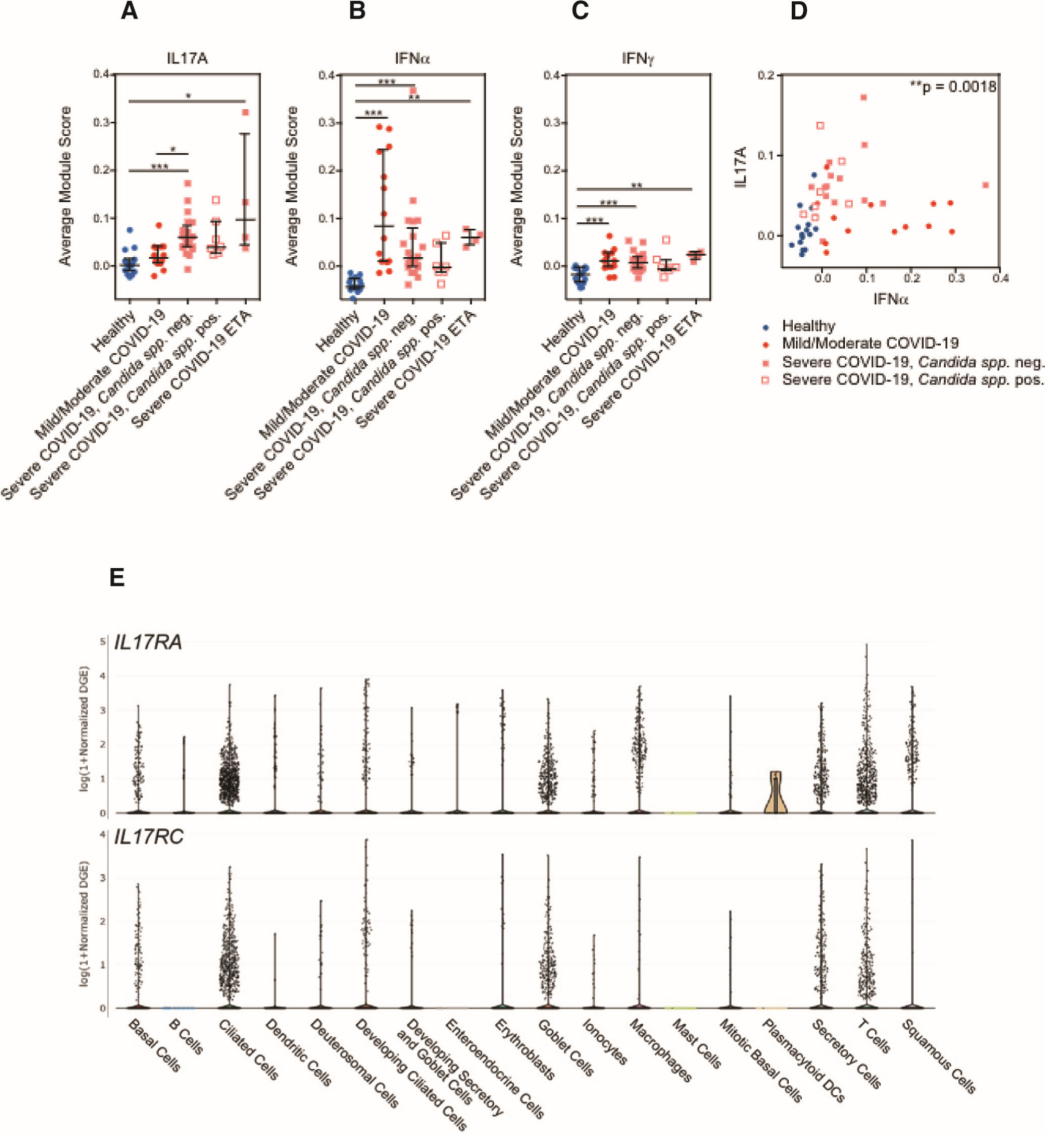
Average module scores in different disease groups in all epithelial cells. (**A–D**) Average gene module scores calculated for each participant, separated by disease group. Module score expression was computed over all epithelial cells. Input module genes derived from *in vitro* stimulation with each labeled cytokine: IL17A (**A**), IFNα (**B**), and IFNγ (**C**). Statistical testing by Kruskal–Wallis test with Dunn’s *post hoc* testing. ^***^P < 0.001, ^**^P < 0.01, and ^*^P < 0.05. Lines represent the mean ± SEM. (**E**) Top: expression of *IL17RA* by cell type. Bottom: expression of *IL17RC* by cell type. Data accessed from https://singlecell.broadinstitute.org/single_cell/study/SCP1289.

Previous findings have reported reductions in type I interferon (27–31) and an increase in IL17 signaling (32–35) in severe COVID-19 patients. To address the impact in the mucosal environment upon exposure to these cytokines, we directly scored epithelial cells for expression of gene signatures indicative of IL17A, IFNα, or IFNγ (Fig. 2A through C). Individuals who developed severe COVID-19 expressed consistently higher abundances of IL17A-induced genes compared to healthy participants or those with mild/moderate COVID-19 (Fig. 2A). Interestingly, we did not detect higher abundances of genes in the IL17A gene module among participants with severe COVID-19 and detectable *Candida* spp. compared to participants with severe COVID-19 without detectable fungal pathogens. Likewise, we did not detect significant differences in levels of interferon-induced signatures between individuals with and without *Candida* spp. detected (Fig. 2B and C). On an individual level, participants whose nasal epithelial cells expressed higher abundances of IFNα-induced genes did not express IL17A-induced genes, and vice versa (Fig. 2D). Together, this suggests that factors beyond *Candida* spp. colonization may be responsible for the induction of IL17A-stimulated genes in the nasal epithelium. However, this underscores that an IL17A-induced epithelial cell state represents a shared feature of individuals who develop severe COVID-19 and is correlated with the absence of robust interferon-induced anti-viral responses.

As expected, the majority of IL17 sources are derived from T cells (Fig. S4A). To further evaluate the relationship between severe COVID-19 and response to IL17 stimulation, we have analyzed our data for the expression of IL17 receptor on epithelial cells. The expression of *IL17RA* and *IL17RC* (which form the heterodimer receptor complex for IL17A) was indeed expressed among nasopharyngeal epithelial cells (Fig. 2E). Ciliated cells, goblet cells, secretory cells, and, to a lesser extent, basal cells were the major epithelial cell types expressing *IL17RA* and *IL17RC*.

### *In vitro* exposure to IL17 in nasal epithelial cells enhances pro-inflammatory responses

**Fig 3:**
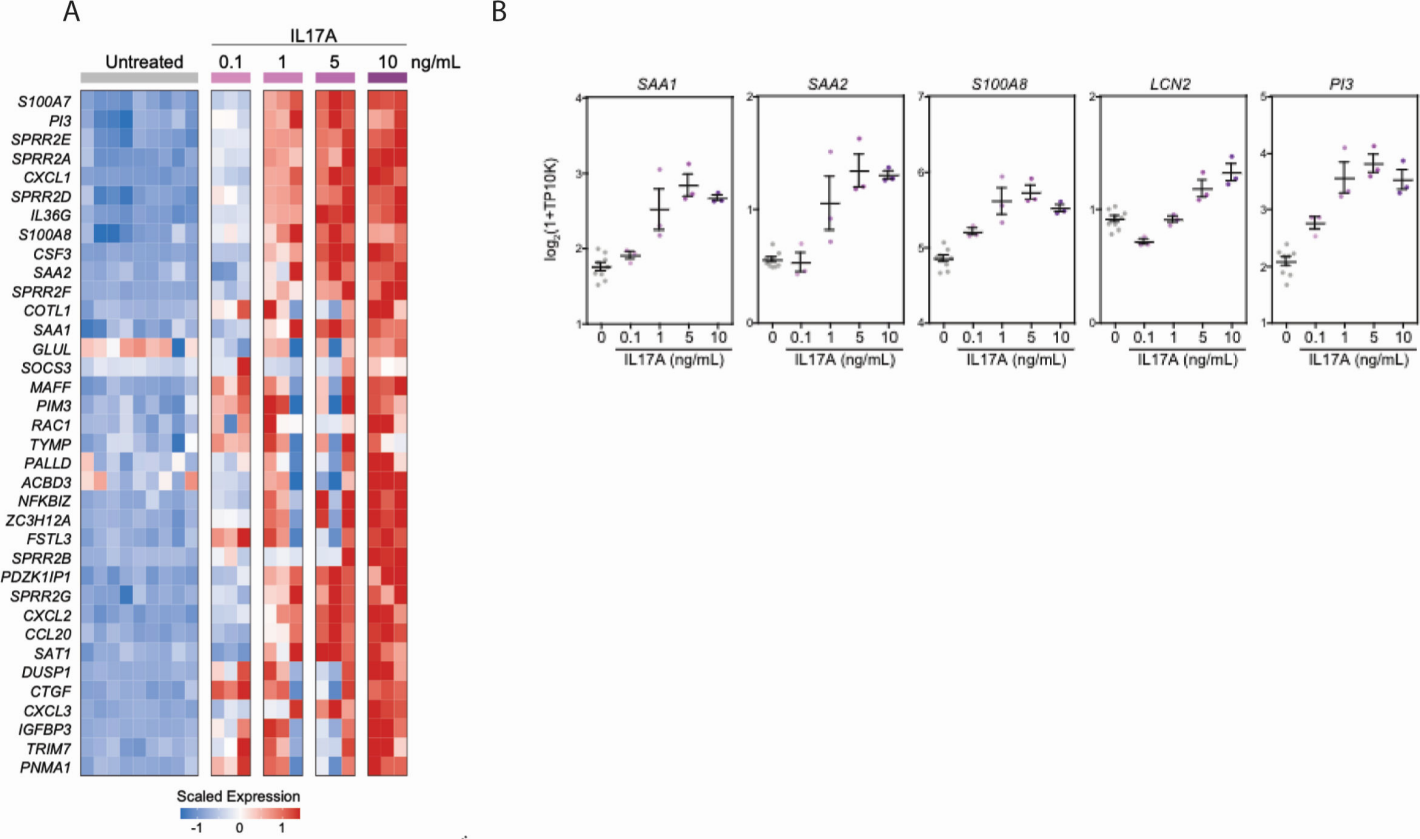
Respiratory epithelial transcriptional signatures following *in vitro* IL17A stimulation and *in vivo* fungal colonization in a severe COVID-19 cohort. (**A**) Heatmap of population RNA-seq data comparing untreated nasal epithelial cells to those treated with increasing concentrations of IL17A as indicated across columns. Genes (rows) with significant differential expression between untreated and IL17A-treated conditions False Discovery Rate [(FDR)-corrected *P* < 0.05)]. (**B**) Expression of select genes following 12-hour stimulation with increasing doses of IL17A. Each gene significantly upregulated following IL17A exposure by likelihood ratio test, FDR-adjusted *P*-value <0.001. Lines represent the mean ± SEM.

Given that *Candida* spp. colonization became a clinically relevant infection among some individuals who developed severe COVID-19, we wondered whether the nasal mucosa of these individuals exhibits evidence for reactive or aberrant IL17 responses. To better define the response of the human nasal epithelium to IL17, we reanalyzed a subset of 15 healthy and 8 severe COVID-19 donors from our previously published population RNA-seq data that reflect gene expression in human nasal epithelial cells following *in vitro* exposure to a range of doses of IL17A (Fig. 3A and B) (36, 37). Across multiple human donors, IL17A exposure led to the upregulation of genes involved in keratinization (*SPRR2E*, *SPRR2F*, and *SPRR2G*); chemoattractant cytokines for lymphocytes, monocytes, and neutrophils (*CCL20*, *CXCL1*, *CXCL2*, and *CXCL3*); and pro-inflammatory factors such as *S100A7* and *S100A8* (Fig. 3A and B) (38–40). IL17A additionally resulted in the dose-dependent induction of serum amyloid A genes *SAA1* and *SAA2* from nasal epithelial cells, which has previously been linked to pathogenic Th17 responses at barrier tissues (41). Using RNA-seq data, we generated consensus gene sets for each tested cytokine that were robust across distinct donors, thereby giving us cell type-specific gene expression modules for IL17A, IFNα, IFNγ, IL1β, and IL4 (Fig. S3A; Table S2).

**Fig 4:**
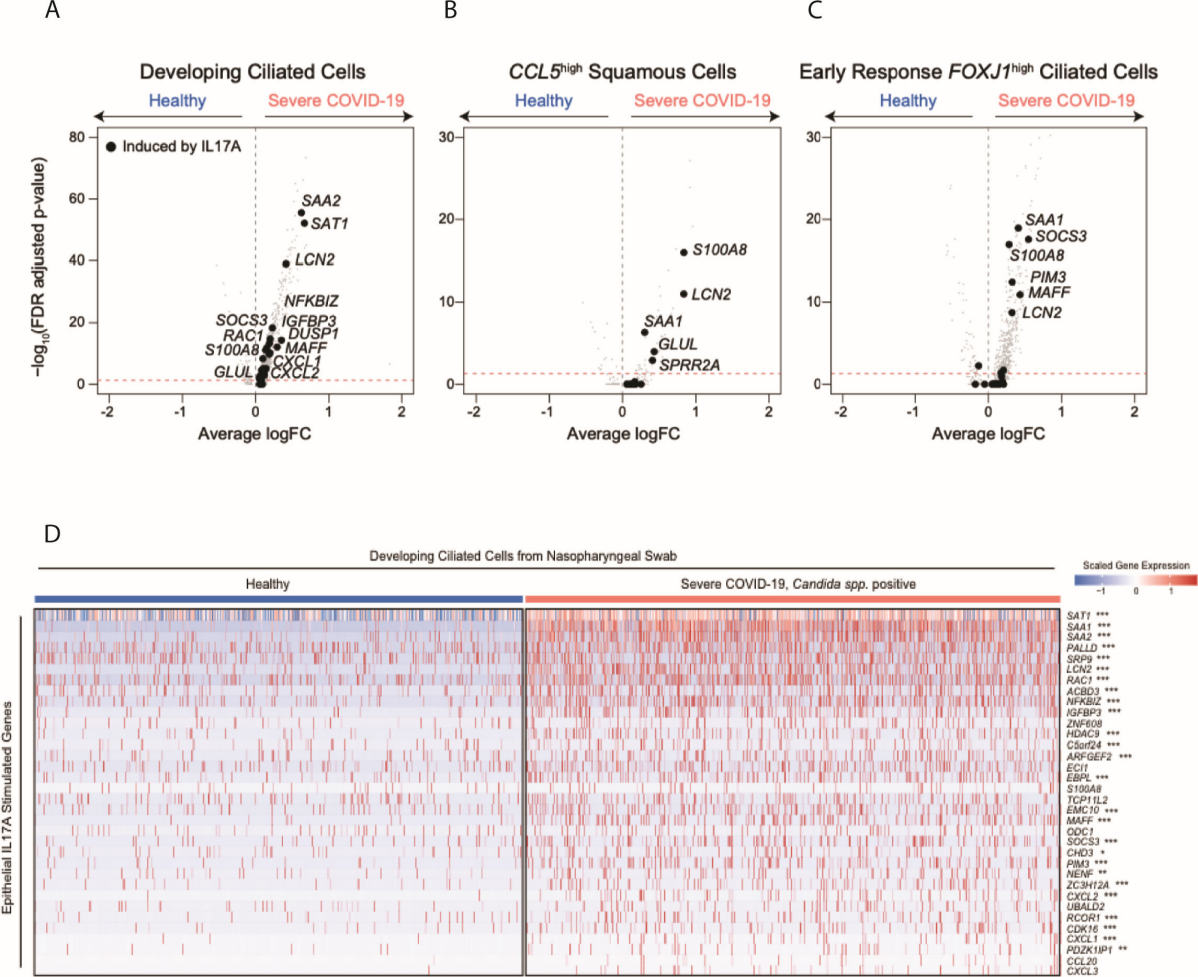
Transcriptional responses of respiratory epithelial cells following *in vitro* exposure to various cytokines (**A–C**). Volcano plots of differentially expressed genes between select epithelial cell types from healthy participants vs those with severe COVID-19: developing ciliated cells (A), *CCL5*^high^ squamous cells (B), and early response *FOXJ1*^high^ ciliated cells (C). Gray points, all genes; black points, genes induced in human nasal epithelial cells following IL17A treatment (as in Fig. 3A). (**D**) Heatmap of IL17A-induced genes among developing ciliated cells from NP swabs. Heatmap colors reflect scaled gene expression: red, higher expression; blue, lower expression. Left columns (blue bar), developing ciliated cells from *n* = 15 healthy participants; right (pink bar), developing ciliated cells from *n* = 8 participants with severe COVID-19 and *Candida* spp. detected. Genes (rows) selected represent genes significantly upregulated among cultured human nasal epithelia cells following *in vitro* exposure to IL17A. Statistical significance comparing healthy-derived vs severe COVID-19, *Candida* spp. positive-derived single cells by likelihood ratio test assuming an underlying negative binomial distribution. ^***^FDR-corrected *P* < 0.001; ^**^FDR < 0.01; ^*^FDR < 0.05.

Next, we returned to the scRNA-seq data from our human COVID-19 cohort and evaluated epithelial cells for transcriptomic signatures consistent with exposure to each cytokine. Compared to epithelial cells isolated from 15 healthy controls, epithelial cells isolated from eight individuals with severe COVID-19 expressed significantly higher levels of genes that were also induced by IL17A exposure *in vitro* (Fig. 4). In particular, IL17A-induced genes *SAA1, SAA2, SAT1, LCN2, S100A8,* and *GLUL* were repeatedly significantly upregulated among diverse epithelial cell subsets in severe COVID-19 (Fig. 4A through C) but were not found to be significantly induced within the nasal epithelia of patients with milder COVID-19 (Fig. S3B). We confirmed that treatment of nasal epithelial cells with other inflammatory signals potentially found within the respiratory epithelium, including IL4 and IL1β, does not appreciably induce these factors, suggesting that the induction of this gene module is a specific downstream effect of IL17 sensing (Fig. S3A).

## DISCUSSION

Our data demonstrate *Candida* spp. colonization of the upper respiratory tract in a significant proportion (38%) of individuals hospitalized with severe COVID-19 in a cohort from the University of Mississippi Medical Center sampled during the summer 2020 COVID-19 peak. Notably, no fungal pathogens were identified among individuals with mild or moderate COVID-19, non-COVID-19 ICU patients, or healthy controls. Fungal reads were detected by NP swabs at early timepoints following hospital admission and within the acute phase of patients’ COVID-19 disease trajectory, suggesting that in some patients, fungal colonization and infection likely occurred prior to hospital admission and in advance of nosocomial exposures. Testing for fungal infection among patients with prolonged intubation and clinical concern for infection is a common occurrence in the intensive care unit. These details provide clinical context and note interesting examples of agreement between the two approaches, but we agree that it does not reflect orthogonal validation.

*Candida* is not typically present in the nares of healthy people, being more readily detected in the oropharynx; thus, the identification of *Candida* from NP swabs of a subset of patients with severe COVID-19 would suggest either that severe COVID-19 predisposes to ectopic colonization in some hosts or alternatively that our methodology is detecting increased fungal abundances derived from the oral mucosa (42). Further direct comparisons of colonization in the mouth and nose of patients with severe COVID-19 will be necessary to clarify this issue (43).

Our analysis additionally unites the use of single-cell transcriptomic technologies in human clinical cohorts with emerging computational approaches for meta-genomic pathogen classification of human samples, all derived from limited cellular material captured on a single NP swab (21, 22). By linking unbiased pathogen detection with single-cell nasal epithelial and immune transcriptional profiles, we have identified specific host behaviors indicative of response to a fungal pathogen and IL17 signaling. For a subset of patients, early detection of *Candida* spp. in the upper respiratory mucosa corresponded to the development of more extensive colonization and clinical concern for secondary fungal pneumonia and/or candidemia. Mounting evidence across various clinical cohorts, including our prior work, suggests that severe COVID-19 arises in individuals with impaired anti-viral immunity (3, 31, 44–50). While there is a paucity of evidence to suggest that type I/III interferon signaling directly restricts fungal colonization, prior *in vitro* studies indicate that IL17 signaling among airway epithelial cells may attenuate cellular responses to type I/III interferon (20, 51–54). Additionally, enhanced virally induced epithelial injury resulting from impaired IFN signaling could facilitate *Candida* colonization (29, 55). Surprisingly, we observe that IL17A-induced gene sets are elevated among epithelial cells from all individuals with severe COVID-19 in our cohort, even those patients without genomic or clinical evidence for coincident fungal infection.

Crucially, our data do not allow us to determine whether our observation of elevated IL17 responses in patients with severe COVID-19 without overt evidence for fungal colonization (i) is the result of colonization below levels of detection in these assays or (ii) suggests that IL17 elevation represents a general phenotype of epithelial cells of patients with severe COVID-19, independent of fungal colonization. Future experiments encompassing longitudinal sampling of patients with COVID-19 could shed additional light on whether variability in the dynamics of IL17 and interferon signaling may underlie *Candida* colonization in the upper airways.

Together, our data suggest that upper respiratory *Candida* colonization and infection represent an underappreciated phenomenon among patients with severe COVID-19. We acknowledge the limited sample number as a limitation to this study, and further research with larger cohorts is warranted to understand the frequency and timing of cooccurring infection with *Candida* spp. and other fungal pathogens following SARS-CoV-2 infection. Our data suggest that dedicated, multi-institutional studies are required to disentangle how clinical and subclinical fungal infections impact patient outcomes during hospitalization for COVID-19 and may hold key insights into determinants of severe respiratory failure for these patients and new strategies for diagnostic or therapeutic intervention.

## MATERIALS AND METHODS

### Participant recruitment and respiratory sampling

Full participant characteristics are provided in a previously published study (3). The UMMC Institutional Review Board approved the study under IRB#2020-0065. All participants or their legally authorized representatives provided written informed consent. Briefly, participants were eligible for inclusion in the COVID-19 group if they were at least 18 years old; had a positive nasopharyngeal swab for SARS-CoV-2 by PCR; had COVID-19-related symptoms including fever, chills, cough, shortness of breath, and sore throat; and weighed more than 110 lb. Participants were eligible for inclusion in the healthy group if they were at least 18 years old, had a current negative SARS-CoV-2 test (PCR or rapid antigen test), and weighed more than 110 lb. COVID-19 participants were classified according to the eight-level ordinal scale proposed by the WHO representing their peak clinical severity and level of respiratory support required. Nasopharyngeal samples and endotracheal aspirate samples were collected by a trained healthcare provider; all processing and handling were carried out as previously described (3, 36).

### scRNA-seq data generation and alignment

Annotated scRNA-seq data were recovered from the Single Cell Portal (see Data Availability), and single-cell annotations were used as described in reference (3). Briefly, data represent aligned scRNA-seq libraries generated using Seq-Well S^3^; libraries were generated using Illumina Nextera XT Library Prep Kits and sequenced on NextSeq 500/550 High Output 75 cycle v2.5 kits to an average depth of 180 million aligned reads per array: read 1: 21 (cell barcode, UMI), read 2: 50 (digital gene expression), and index 1: 8 (N700 barcode) (56). Libraries were aligned using STAR within the Drop-Seq Computational Protocol (https://github.com/broadinstitute/Drop-seq) and implemented on Cumulus (https://cumulus.readthedocs.io/en/latest/drop_seq.html, snapshot 9, default parameters) (57, 58). As previously described, data were aligned using a custom reference, which combined human GRCh38 (from CellRanger version 3.0.0, Ensembl 93) and SARS-CoV-2 RNA genomes (3, 59).

### Meta-transcriptomic pathogen classification

To identify codetected microbial taxa present in the cell-associated or ambient RNA of nasopharyngeal swabs, we used the Kraken2 software implemented using the Broad Institute viral-ngs pipelines on Terra (https://github.com/broadinstitute/viral-pipelines/tree/master) (21, 22). A previously published reference database included human, archaea, bacteria, plasmid, viral, fungi, and protozoa species and was constructed on 5 May 2020, therefore including sequences belonging to the novel SARS-CoV-2 virus. Inputs to Kraken2 were the following: kraken2_db_tgz = “gs://pathogen-public-dbs/v1/kraken2-broad-20200505.tar.zst,” krona_taxonomy_db_kraken2_tgz = “gs://pathogen-public-dbs/v1/krona.taxonomy-20200505.tab.zst,” ncbi_taxdump_tgz = “gs://pathogen-public-dbs/v1/taxdump-20200505.tar.gz,” trim_clip_db = “gs://pathogen-public-dbs/v0/contaminants.clip_db.fasta,” and spikein_db = “gs://pathogen-public-dbs/v0/ERCC_96_nopolyA.fasta.” Results were collected using the merge_metagenomics tool (https://viral-pipelines.readthedocs.io/en/latest/merge_metagenomics.html), and analysis and visualization of each sample’s metagenomic alignments were implemented in Prism (v6) or R [v4.0.2; packages ggplot2 (v3.3.2), Seurat (v3.2.2), and ComplexHeatmap (v2.7.3)]. All classification data are included in Table S1.

### Human nasal epithelial cell response to *in vitro* cytokine exposure

Gene lists representing human nasal epithelial cell responses to various exogenous cytokines *in vitro* are derived from previously published population RNA-seq data (36, 37). Briefly, human nasal epithelial basal cells from two donors were stimulated *in vitro* with 0.1–10 ng/mL IFNα, IL17A, IFNγ, IL1β, or IL4 for 12 hours. Following stimulation, cells were lysed, and bulk RNA-seq libraries were generated using the SMART-Seq2 protocol (60). We identified epithelial gene sets induced by each cytokine independently by testing for differentially expressed genes compared to matched, untreated nasal epithelial samples (*n* = 10). Differential expression testing was carried out using a likelihood ratio test assuming a negative binomial distribution, implemented with the Seurat (v3.1.5) FindAllMarkers function (test.use = “negbinom”). We considered genes as differentially expressed with an FDR-adjusted *P*-value <0.05 (61).

To score for cytokine-specific gene expression among COVID-19 or healthy scRNA-seq samples, we first subsetted our scRNA-seq data to only epithelial cells using “coarse” cell types, as defined by a cell typing procedure carried out in prior publication (3). Coarse cell type groups that were included in the analysis include “ciliated cells,” “developing ciliated cells,” “secretory cells,” “goblet cells,” “ionocytes,” “deuterosomal cells,” “squamous cells,” “basal cells,” “mitotic basal cells,” and “developing secretory and goblet cells.” We calculated module scores over all epithelial cells using the Seurat function AddModuleScore with default inputs. The average module score for each NP or ETA sample was utilized as a representative measure of epithelial behavior for each participant, as represented in Fig. 2A through D.

### scRNA-seq analysis of differential expression

To compare gene expression between cells from distinct disease groups (e.g., healthy vs severe COVID-19, *Candida* spp.), we employed a likelihood ratio test assuming a negative binomial distribution as described above [using Seurat FindAllMarkers function (test.use = “negbinom”)] (61). We considered genes as differentially expressed with an FDR-adjusted *P*-value <0.001 and log fold change >0.25. Results from select “detailed” cell types, as defined and previously reported by Ziegler et al., are represented in Fig. 4A through C (3). Full results of differential expression as represented in Fig. 4A through C can be found in supplementary tables.

### Statistical testing

All statistical tests were implemented in R (v4.0.2) or Prism (v6) software. Specific statistical tests, *P*-values, *n*, and all summary statistics are provided in Results, figure legends, and/or figure panels.

## Data Availability

All scRNA-seq and RNA-seq data analyzed in this study are publicly available from prior manuscripts. Aligned and annotated scRNA-seq data can be downloaded via the Single Cell Portal, study SCP1289. Aligned TPM-normalized RNA-seq data can be downloaded via the Single Cell Portal, study SCP822. Results from Kraken2 metagenomic classification are reported in Table S1.

## SUPPLEMENTAL MATERIAL

Supplemental figures spectrum.03516-23-s0001.pdf Fig. S1 to S4.

Table S1 spectrum.03516-23-s0002.xlsx Metatranscriptomic data output from kraken2.

Table S2 spectrum.03516-23-s0003.xlsx Gene sets.

Table S3 spectrum.03516-23-s0004.xlsx Control vs COVID in epithelial cells.

ASM does not own the copyrights to Supplemental Material that may be linked to, or accessed through, an article. The authors have granted ASM a non-exclusive, world-wide license to publish the Supplemental Material files. Please contact the corresponding author directly for reuse.
