# Supplementary material for: An enhanced IL17 and muted type I interferon nasal epithelial cell state characterizes severe COVID-19 with fungal coinfection
Source: Microbiol Spectr. 2024 Apr 30;12(6):e03516-23. doi: 10.1128/spectrum.03516-23 (PMC11237666; doi:10.1128/spectrum.03516-23)
Supplement: Supplemental figures — Fig. S1 to S4. [file spectrum.03516-23-s0001.pdf]

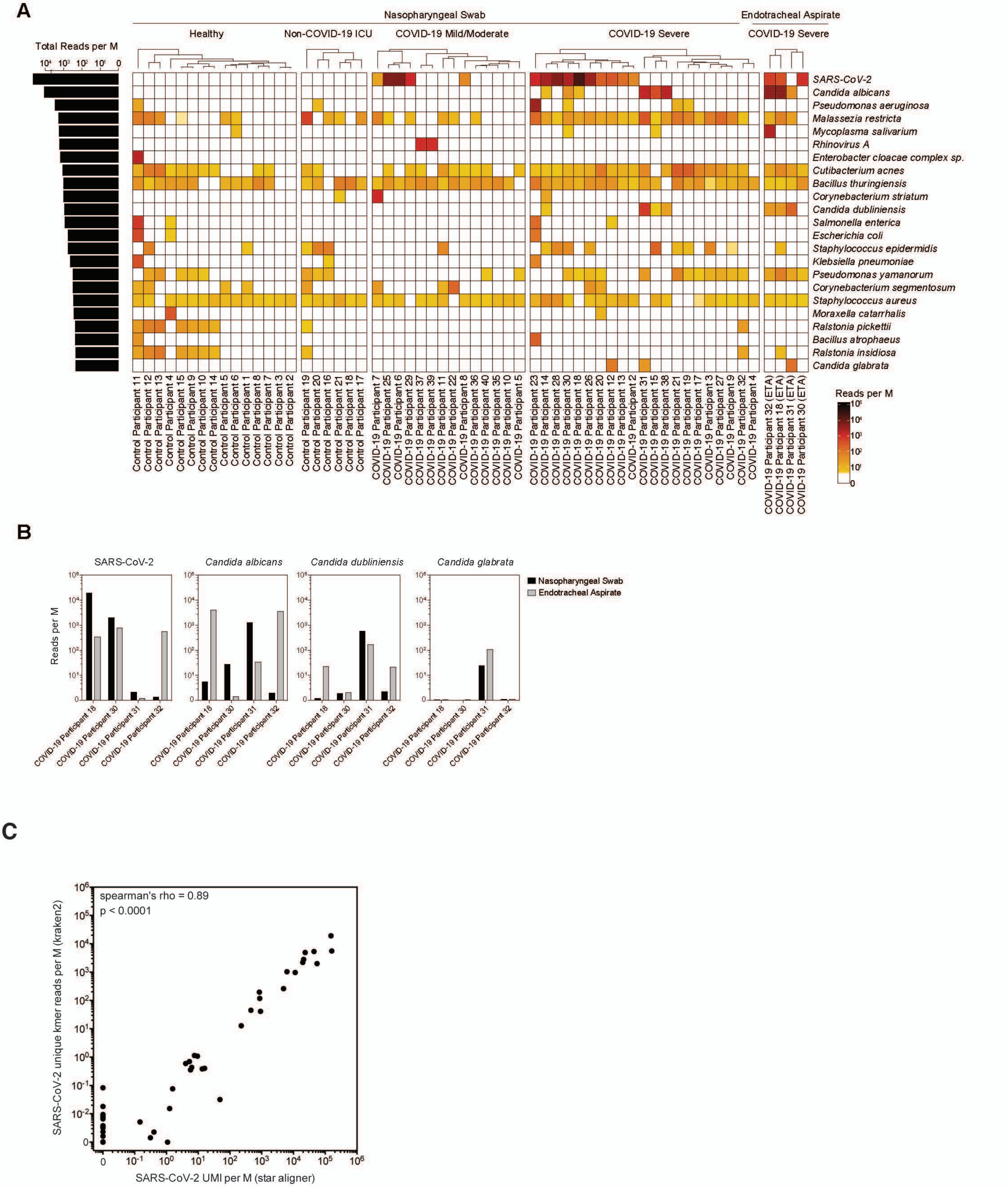

Supplemental Figure 1. Meta-transcriptomic classification of reads from nasopharyngeal swabs and endotracheal aspirates

A. Heatmap of top detected microbes (rows) across all samples (columns). Bar plot on left: total classified reads per million (M) for each microbe listed along heatmap rows. Heatmap colors represent total classified reads per M, dark red: higher abundance, yellow: lower abundance, white: < 10 reads per M.

B. SARS-CoV-2 and Candida spp. reads detected from matched nasopharyngeal (NP, black bars) and endotracheal aspirate (ETA, grey bars) samples from the same participants.

C. Abundance of UMI aligning to SARS-CoV-2 RNA for each patient. Y axis: reads detected by metagenomic classification of SARS-CoV-2 RNA. N=56 participants, both Healthy and COVID-19.

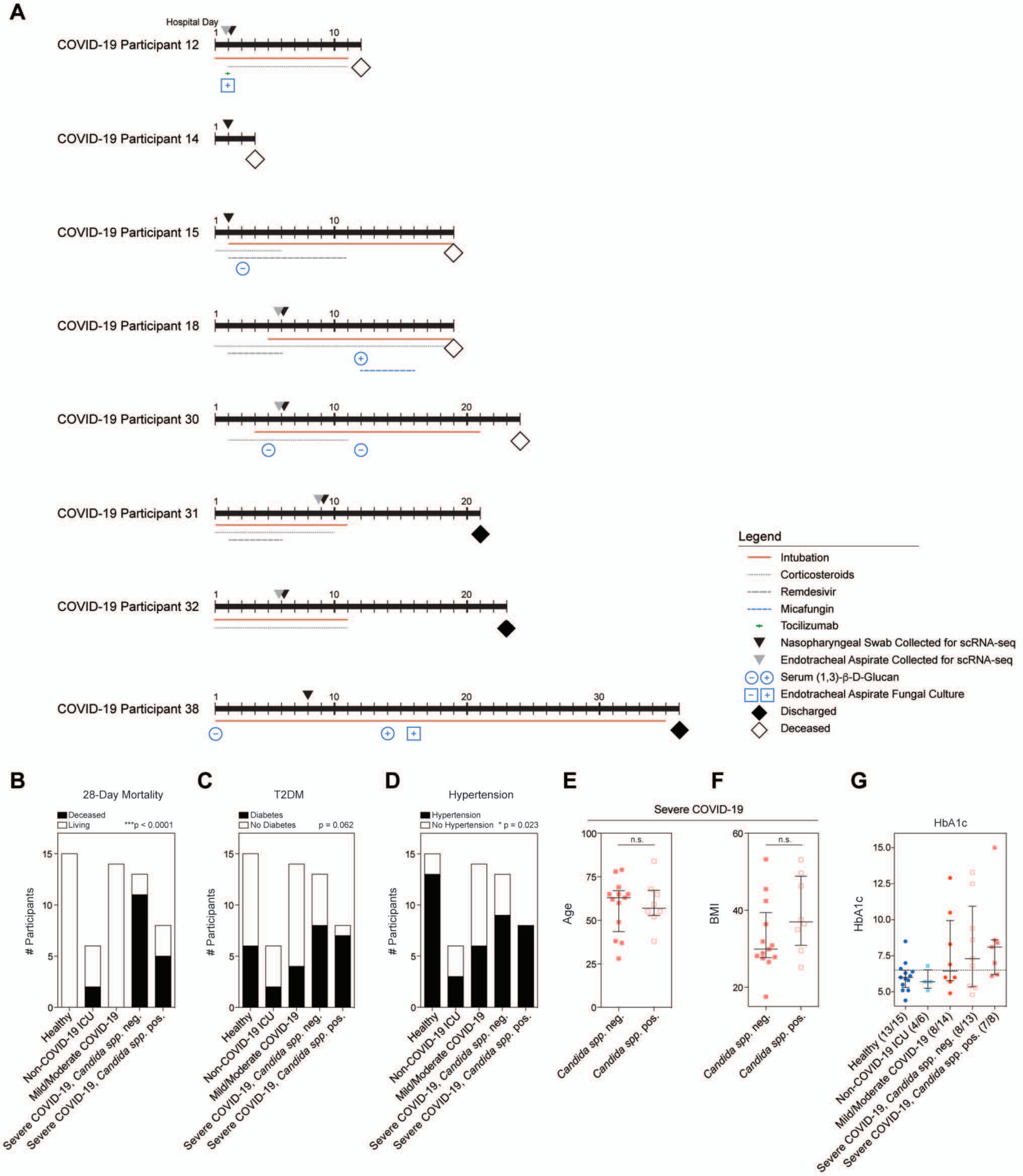

Supplemental Figure 2. Participant characteristics and clinical course

A. Hospital timelines for 8 participants with *Candida spp*. detected from scRNA-seq NP or ETA samples.

B.-D. Select categorical demographic and clinical information from study participants by disease cohort: 28-day mortality (**B**), type 2 diabetes mellitus (T2DM) (**C**), and chronic hypertension (**D**). Statistical testing by multi-group chi-square test, significance result is reported above each plot.

E.-F. Select continuous demographic and clinical information among participants with severe COVID-19, separated by detection of *Candida spp.*: age (**E**) and BMI (**F**). Statistical testing using student's t-test. Lines represent median +/- interquartile range.

G. Hemoglobin A1c (HbA1c) for each study participant by disease cohort. Dashed line at HbA1c 6.5%. Group differences non-significant by Kruskal-Wallis test. Numbers in parenthesis reflect patients per group with available data in medical record.

A

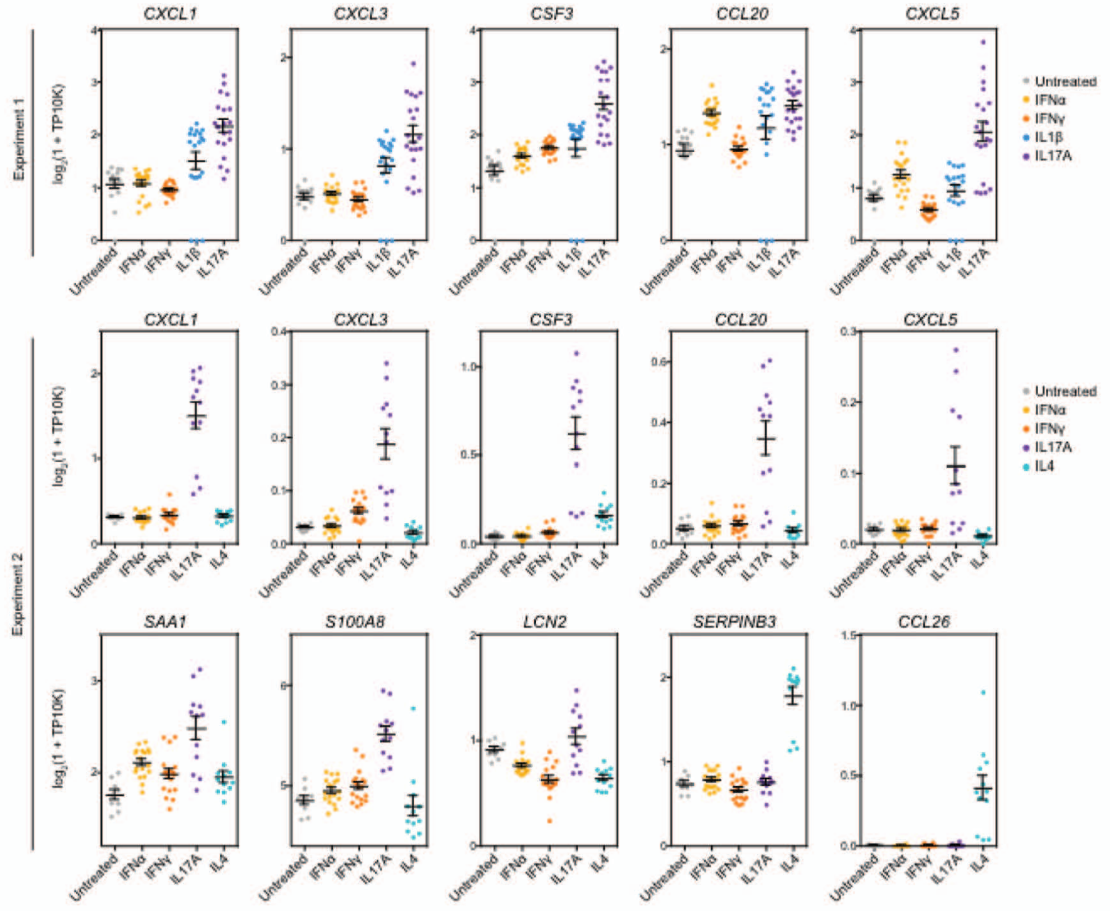

B

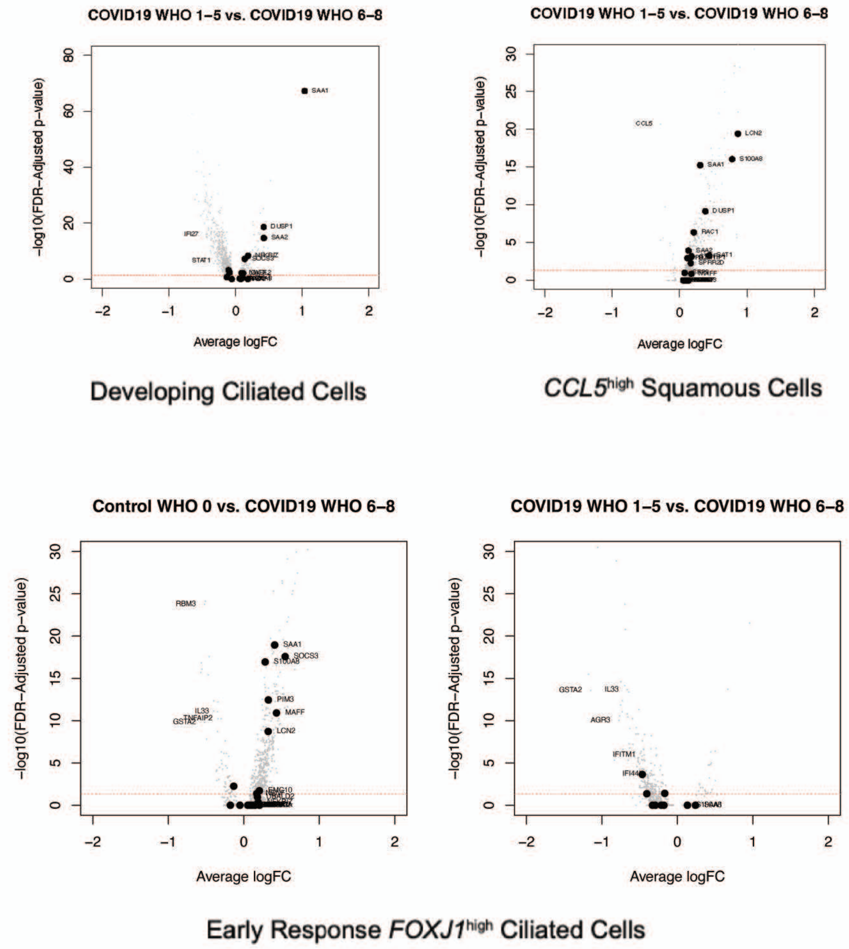

Supplemental Figure 3. Expression of select genes among human nasal basal cells by stimulation condition.

A. Expression of select genes among human nasal basal cells presented on by different cytokine simulation condition across 2 separate experiments.

B. Volcano plots of differential gene expression in selected epithelial cells comparing mild to severe COVID-19.

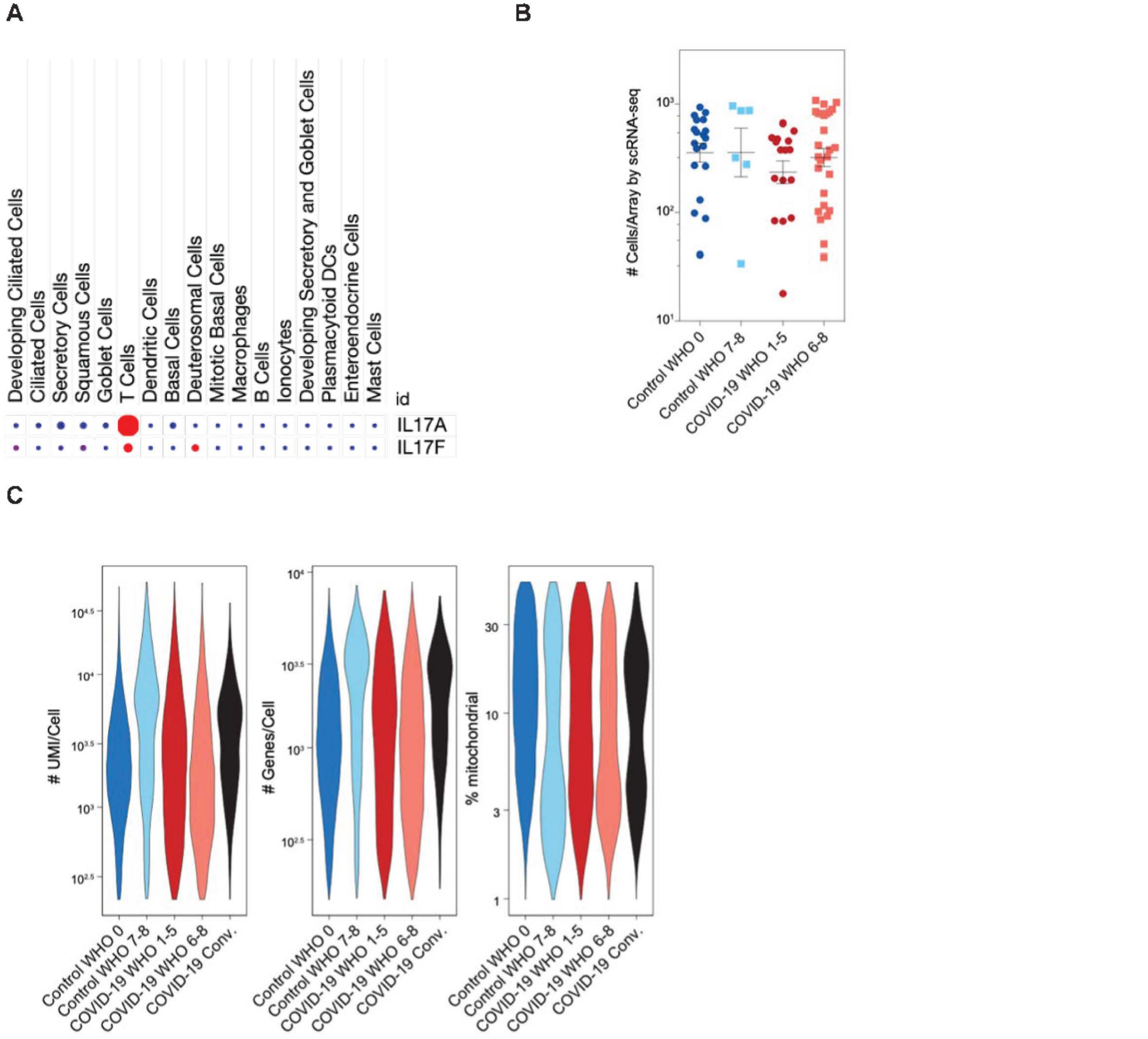

Supplemental Figure 4. Quality control analysis

A. Dot plot representing expression abundance of IL17A and IL17F according to cell type across all patients. Dot size corresponds to % of cell type expressing each gene.

B. Number of high-quality cells/array recovered for single-cell RNA-seq by disease group. Statistical testing by Kruskal-Wallis test ($p = 0.37$) with Dunn's post hoc testing, all $p > 0.05$. Dark blue circles: Healthy ("Control WHO 0"); light blue squares: Non-COVID-19 ICU ("Control WHO 7-8"); red circles: Mild/Moderate COVID-19 ("COVID-19 WHO 1-5); pink squares: Severe COVID-19 ("COVID-9 WHO 6-8").

C. Single-cell quality metrics by group (after filtering for low-quality cells). Left: number of UMI per cell. Center: number of genes per cell. Right: % mitochondrial mapping per cell. Blue: Healthy ("Control WHO 0"); light blue: Non-COVID-19 ICU ("Control WHO 7-8"); red: Mild/Moderate COVID-19 ("COVID-19 WHO 1-5); pink: Severe COVID-19 ("COVID-19 WHO 6-8"); black: COVID-19 convalescent (excluded from this study).
